# Association between baseline blood pressure and the incidence of lenvatinib‐induced hypertension in patients with thyroid cancer

**DOI:** 10.1002/cam4.6644

**Published:** 2023-10-30

**Authors:** Yuma Shibutani, Kazuko Tajiri, Shinya Suzuki, Tomohiro Enokida, Atsunobu Sagara, Susumu Okano, Takao Fujisawa, Fumiaki Sato, Tetsuro Yumoto, Motohiko Sano, Toshikatsu Kawasaki, Makoto Tahara

**Affiliations:** ^1^ Department of Pharmacy National Cancer Center Hospital East Kashiwa Japan; ^2^ Hoshi University School of Pharmacy and Pharmaceutical Sciences Shinagawa Japan; ^3^ Department of Cardiology National Cancer Center Hospital East Kashiwa Japan; ^4^ Tsukuba Life Science Innovation Program (T‐LSI), School of Integrative and Global Majors (SIGMA) University of Tsukuba Tsukuba Japan; ^5^ Department of Head and Neck Medical Oncology National Cancer Center Hospital East Kashiwa Japan

**Keywords:** antihypertensive treatment, cardio‐oncology, onco‐cardiology, tyrosine kinase inhibitor, VEGF inhibitor

## Abstract

**Background:**

Hypertension is the most frequently occurring adverse event of lenvatinib, recognized relatively early in its course. However, the trend in blood pressure after the initiation of lenvatinib and the outcomes with antihypertensive treatment are unclear. This study aimed to clarify the association between baseline blood pressure and the incidence of lenvatinib‐induced hypertension in patients with thyroid cancer.

**Method**s**:**

This retrospective study included 65 patients without hypertension at the time of lenvatinib initiation. Patients were divided into two groups: those who developed hypertension grade ≥3 (HTN group) and those who did not develop hypertension grade ≥3 (non‐HTN group).

**Results:**

Of the 65 patients, 46 (71%) developed hypertension grade ≥3. In both HTN and non‐HTN groups, blood pressure significantly increased the day after lenvatinib initiation. There was no significant difference in the elevated values of both the changes in systolic blood pressure (ΔSBP) and diastolic blood pressure (ΔDBP) between the two groups, with an average increase of 20 mmHg in SBP and 13 mmHg in DBP from baseline. The median (range) time to the onset of hypertension grade ≥3 was 2 days (1–12 days). In the multivariable analysis, patients with normal (SBP 120–129 mmHg and/or DBP 80–84 mmHg) or high‐normal baseline blood pressure (SBP 130–139 mmHg and/or DBP 85–89 mmHg) were at higher risk of developing hypertension grade ≥3 than those with optimal baseline blood pressure (SBP <120 mmHg and DBP <80 mmHg) (odds ratio [OR], 5.07; 95% confidential interval [CI] 1.09–23.54 and OR, 7.48; 95% CI, 1.67–33.51, respectively).

**Conclusions:**

Lenvatinib‐induced hypertension appears the day after administration, and higher baseline blood pressure is a significant risk factor for developing hypertension grade ≥3. In cases of increased blood pressure with lenvatinib, early initiation of antihypertensives may prevent treatment interruption due to hypertension and maintain the therapeutic intensity of lenvatinib.

## 1 INTRODUCTION

The incidence of thyroid cancer has been increasing over the past 30 years, with differentiated thyroid cancer (DTC) being the most common histologic type.^1^, ^2^ The standard treatment for DTC is surgery, often in combination with radioiodine (RAI).^3^, ^4^ In Phase 3 of the Study of Lenvatinib in Differentiated Cancer of the Thyroid (SELECT) trial, lenvatinib significantly prolonged progression‐free survival (PFS) to 18.3 months in patients with RAI‐refractory (RR)‐DTC, compared to 3.6 months for placebo; thus, the use of lenvatinib is recommended for patients with RR‐DTC.^5^, ^6^

Lenvatinib is a multikinase inhibitor that inhibits vascular endothelial growth factor (VEGF) receptors 1 to 3 and other angiogenic and oncogenic receptor tyrosine kinases.^7^, ^8^, ^9^ As with other VEGF and VEGF receptor inhibitors, hypertension is one of the most frequently occurring adverse events with lenvatinib. In the SELECT trial, the rate of occurrence of hypertension was 67.8% for all grades and 41.8% for grade ≥3.^5^, ^10^ As hypertension is one of the dose‐limiting toxicities of lenvatinib, the incidence of grade ≥3 hypertension leads to dose reductions or interruptions.^5^, ^11^ According to a post hoc analysis of the SELECT trial, patients who required a longer interruption (≥10% of the total treatment duration) had a shorter PFS compared to patients who required a shorter interruption (<10%).^12^ The group with longer dose interruptions had a lower median dose intensity of lenvatinib (14.6 mg/day) compared to the group with shorter interruptions (20.1 mg/day).^12^ These findings highlight the significance of maintaining the dose intensity of lenvatinib in patients with RR‐DTC. Furthermore, in thyroid cancer, as in thymic carcinoma, high‐dose lenvatinib (24 mg/day) is recommended and has been associated with a higher incidence of hypertension than in other carcinomas treated with low to moderate doses of lenvatinib.^5^, ^13^, ^14^, ^15^

In the SELECT trial, 47% of lenvatinib‐treated patients experienced their first episode of hypertension during the first cycle of treatment.^16^ Thus, as lenvatinib treatment causes hypertension with high frequency, close monitoring and regulation of blood pressure during lenvatinib treatment is important, especially in patients with thyroid cancer; however, the details of the changes in blood pressure and risk factors for the development of hypertension are not fully understood.

In our hospital, when initiating lenvatinib, patients are hospitalized for approximately 2 weeks for the purpose of managing adverse events before moving to the outpatient setting. To clarify the association between baseline blood pressure and the incidence of lenvatinib‐induced hypertension in patients with thyroid cancer, we analyzed the data of patients who were administered lenvatinib and hospitalized for the purpose of managing adverse events to examine the details of blood pressure changes during treatment and the risk factors for the development of hypertension grade ≥3.

## 2 METHODS

### 2.1 Subjects

We used an institutional database to identify all patients with thyroid cancer who were treated with lenvatinib and completed an initial inpatient treatment phase from May 1, 2015, to October 31, 2022. This study was approved by the review board of the National Cancer Center (research project no. 2022‐115). The requirement of informed consent was waived because this study was conducted according to a retrospective chart review protocol.

Among the 73 patients with thyroid cancer treated with lenvatinib, a total of 8 patients who had uncontrolled hypertension (systolic blood pressure [SBP] ≥140 mmHg or diastolic blood pressure [DBP] ≥90 mmHg) at the start of lenvatinib administration were excluded from the study to examine the effect of lenvatinib on blood pressure under stable conditions. The remaining 65 patients who were administered 24 mg/day of lenvatinib in the hospital setting for thyroid cancer were included in the final analysis.

### 2.2 Study design

This was a single‐center retrospective study, and all data were extracted from medical records. Lenvatinib was started orally at 24 mg daily in all patients, with dose reduction or interruption at the physician's discretion in the event of unacceptable adverse events. Therefore, there was no unified protocol for dose reduction or interruption among attending physicians. Hypertension was graded with the Common Terminology Criteria for Adverse Events version 5.0. Trend in blood pressure changes with lenvatinib treatment, incidence of hypertension grade ≥3 (SBP ≥160 or DBP ≥100 mmHg), and antihypertensive treatment were evaluated early after lenvatinib initiation. Patients were classified into two groups: the hypertension group (HTN), consisting of patients who developed hypertension grade ≥3 while receiving lenvatinib, and the non‐hypertension group (non‐HTN), consisting of patients who did not develop grade ≥3 hypertension.

The study period was 14 days, which was the length of hospitalization for the purpose of adverse event management, and detailed data on blood pressure changes from the start of lenvatinib administration after admission until withdrawal or dose reduction due to an adverse event or discharge from hospital were evaluated. Blood pressure was measured by nurses at least thrice a day as part of blood pressure assessments during hospitalization. During that blood pressure monitoring, if SBP was ≥160 and/or DBP was ≥100 mmHg, blood pressure was re‐measured after 30 min under rest instructions.

Baseline blood pressure was defined as the average of values measured from the time of admission until lenvatinib was initiated. The median number of blood pressure measurements from admission to initiation of lenvatinib was 3 (interquartile range: 3–4). The mean daily blood pressure during lenvatinib treatment was the average blood pressure measured multiple times during the day. The change in blood pressure (ΔSBP and ΔDBP) was determined by subtracting the pre‐lenvatinib (baseline) blood pressure from the values measured at each time point after lenvatinib treatment. Baseline blood pressure was classified into three categories according to the 2018 ESC/ESH Clinical Practice Guidelines for the Management of Atrial Hypertension: optimal (SBP <120 mmHg and DBP <80 mmHg), normal (SBP 120–129 mmHg and/or DBP 80–84 mmHg), and high‐normal (SBP 130–139 mmHg and/or DBP 85–89 mmHg).^17^

As for patient characteristics, history of hypertension was defined as a diagnosis of hypertension and receiving antihypertensive treatment prior to lenvatinib initiation. We also evaluated patients who had previously been diagnosed with diabetes, dyslipidemia, cardiovascular disease (CVD), or stroke prior to initiation of lenvatinib. History of CVD included arrhythmias, such as atrial fibrillation, coronary artery disease, myocardial infarction, chronic heart failure, and severe valvular disease diagnosed prior to lenvatinib initiation. Performance status was assessed using the Eastern Cooperative Oncology Group scale. The estimated glomerular filtration rate (eGFR) was calculated using the estimation equation for Japanese patients.^18^ The eGFR was calculated from serum creatinine, adjusted for age and sex, using the following formula: (eGFR [mL/min/1.73 m^2^] = 194 × age‐0.287 × serum creatinine‐1.094 × [0.739 for women]).

### 2.3 Statistical analysis

Continuous variables are presented as the mean ± standard deviation or the median and interquartile range based on their distributions with or without normality. Categorical variables are presented as numbers and percentages. Normality was verified using the Shapiro–Wilk test. Continuous variables were compared using the unpaired Student's *t*‐test or Mann–Whitney *U*‐test. Categorical variables were compared using Pearson's chi‐squared test or Fisher's exact test. In addition, logistic regression analysis was used in the multivariate analysis. For factor selection, after all factors were introduced, two factors were extracted from those with small *p*‐values using the variable reduction method. A *p* < 0.05 was considered statistically significant. Statistical analysis was performed using SPSS statistics ver. 28.0 (IBM).

## 3 RESULTS

### 3.1 Patient characteristics

Of the 65 patients analyzed, 46 (71%) developed hypertension grade ≥3 while receiving lenvatinib (HTN group), while 19 (29%) did not (non‐HTN group; Table 1). There were no significant differences in baseline characteristics between the two groups, except for baseline blood pressure. Baseline SBP and DBP were significantly higher in the HTN group than in the non‐HTN group (SBP, *p* = 0.004; DBP, *p* = 0.005). Baseline blood pressure was within the normal range (SBP <140 mmHg and DBP <90 mmHg) in all patients; however, the HTN group had the highest number of patients with high‐normal blood pressure, and the non‐HTN group had the highest number of patients with optimal blood pressure (*p* = 0.014).

**TABLE 1:** Patient characteristics.

	Non‐HTN (*N* = 19)	HTN (*N* = 46)	*p*‐Value
Female sex	9 (47)	32 (69)	0.092
Age (years)	73 (56–84)	71 (43–82)	0.348
ECOG PS			
0	13 (68)	26 (56)	0.460
1	5 (26)	19 (41)	
2	1 (5)	1 (2)	
3 or 4	0 (0)	0 (0)	
Histological type; PTC/FTC/PDTC/ATC	10/5/0/4	30/8/1/7	0.567
Baseline SBP (mmHg)	115.1 ± 14.1	126.2 ± 9.1	0.004
Baseline DBP (mmHg)	68.9 ± 7.8	75.1 ± 7.8	0.005
Blood pressure group			
Optimal (SBP <120 and DBP <80 mmHg)	11 (57)	10 (21)	0.014
Normal (SBP 120–129 and/or DBP 80–84 mmHg)	4 (21)	13 (28)	
High‐normal (SBP 130–139 and/or DBP 85–89 mmHg)	4 (21)	23 (50)	
eGFR (mL/min/1.73 m^2^)	75.7 (44.0–124.5)	64.5 (41.0–121.6)	0.099
eGFR <60 (mL/min/1.73 m^2^)	4 (21)	14 (30)	0.442
Obesity (BMI ≥25 kf/m^2^)	4 (21)	14 (30)	0.442
Smoking	10 (52)	15 (32)	0.131
Medical history			
Hypertension	10 (52)	22 (47)	0.724
Diabetes	5 (26)	9 (19)	0.384
Dyslipidemia	4 (21)	14 (30)	0.442
CVD and/or stroke	4 (21)	9 (19)	0.569
Antihypertensive therapy	10 (52)	19 (41)	0.403
Medication			
CCB	6 (31)	12 (26)	0.653
ACEI/ARB	8 (42)	12 (26)	0.203
β blockers	1 (5)	1 (2)	0.502
α blockers	1 (5)	0 (0)	0.292
Diuretics	1 (5)	0 (0)	0.292

*Note*: Data are expressed as *n* (%), mean ± SD or median (interquartile range). ACEI/ARB: angiotensin‐converting enzyme inhibitor/angiotensin II receptor blocker.

Abbreviations: ATC, anaplastic thyroid cancer; BMI, body mass index; CCB, calcium channel blocker; CVD, cardiovascular disease; DBP, diastolic blood pressure; ECOG PS, Eastern Cooperative Oncology Group Performance Status; eGFR, estimated glomerular filtration rate; FTC, follicular thyroid cancer; HTN, hypertension; PDTC, poorly differentiated thyroid cancer; PTC, papillary thyroid cancer; SBP, systolic blood pressure.

### 3.2 Blood pressure trends after starting lenvatinib

**FIGURE 1:**
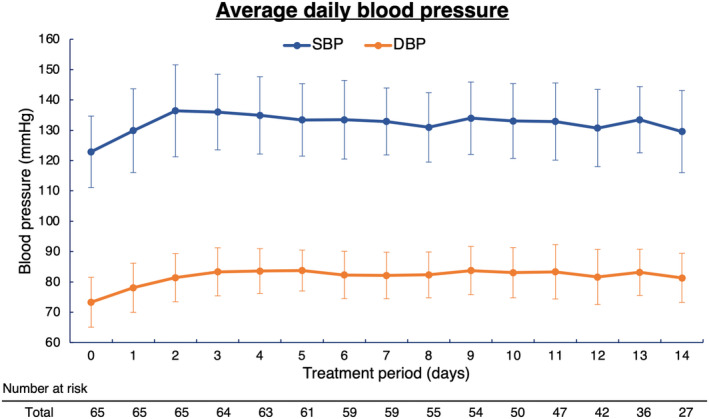
Changes in daily blood pressure after lenvatinib treatment. Daily trends in systolic blood pressure (SBP) and diastolic blood pressure (DBP) are shown.

**FIGURE 2:**
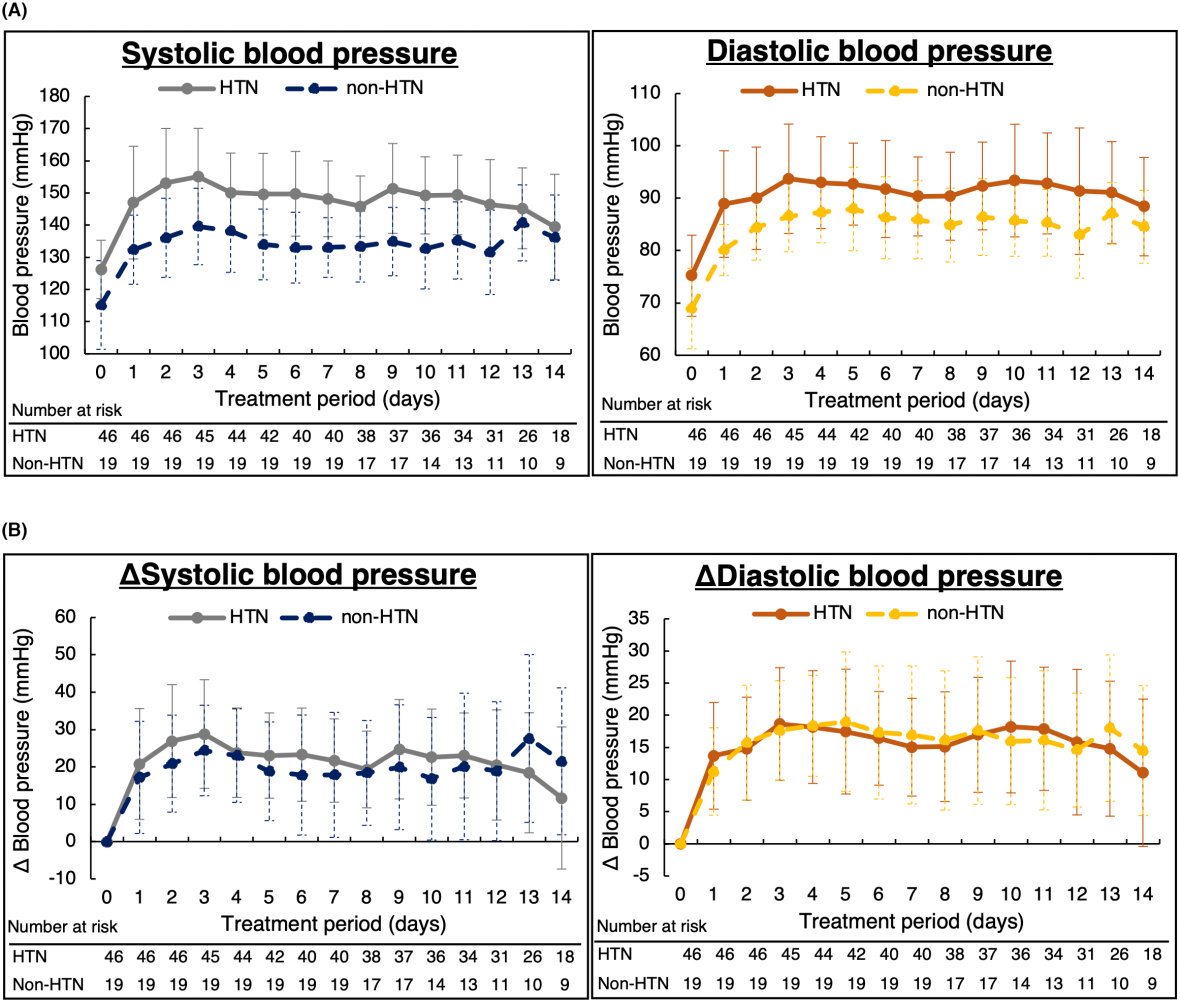
Comparisons of blood pressure trends after lenvatinib administration. (A) Changes in blood pressure in each group. (B) Changes in blood pressure from baseline in each group.

The blood pressure trend of SBP and DBP in all 65 patients showed an increase in SBP and DBP from Day 1 after the start of lenvatinib and a leveling off trend after Day 3 (Figure 1). The median (range) time to onset of hypertension grade ≥3 was 2 (1–12) days. Daily trends in SBP and DBP were different between the HTN and non‐HTN groups (Figure 2A). However, ΔSBP and ΔDBP were similar at each time point between the two groups (Figure 2B). We also examined blood pressure trends separately for patients who were taking antihypertensive medications prior to lenvatinib and for those who were not. As shown in Figures S1 and S2, patients who were taking antihypertensive drugs prior to lenvatinib and those who were not had similar blood pressure trends. In both patient groups, those who developed grade ≥3 hypertension had higher blood pressure prior to lenvatinib administration. The maximal values of daily SBP and DBP were significantly higher in the HTN group, but the values of the difference between maximal and baseline blood pressure did not differ between the two groups (Table 2). No patients interrupted lenvatinib treatment due to hypertension during the study period. During outpatient treatment after the observation period of this study, seven patients discontinued or reduced lenvatinib treatment because of hypertension (interrupted or reduced lenvatinib dose: 24 mg [*n* = 1], 20 mg [*n* = 4], and 14 mg [*n* = 2]).

**TABLE 2:** Comparison of average blood pressure at each time point.

	Non‐HTN (*N* = 19)	HTN (*N* = 46)	*p*‐Value
SBP (mmHg)			
Baseline	115.1 ± 14.1	126.2 ± 9.1	0.004
Maximum value after lenvatinib initiation	137.9 ± 9.7	149.4 ± 9.6	<0.001
ΔSBP (maximum‐baseline)	22.7 ± 12.2	23.2 ± 10.8	0.876
1 week after lenvatinib initiation	124.0 ± 8.9	137.0 ± 9.5	<0.001
2 weeks after lenvatinib initiation	127.4 ± 14.3	130.6 ± 13.8	0.586
DBP (mmHg)			
Baseline	68.9 ± 7.8	75.1 ± 7.8	0.005
Maximum value after lenvatinib initiation	85.8 ± 4.3	91.1 ± 7.2	<0.001
ΔDBP (maximum‐baseline)	16.9 ± 7.7	15.9 ± 7.7	0.645
1 week after lenvatinib initiation	79.1 ± 6.6	83.5 ± 7.8	0.038
2 weeks after lenvatinib initiation	77.7 ± 6.4	83.1 ± 8.6	0.109

Abbreviations: DBP, diastolic blood pressure; HTN, hypertension; SBP, systolic blood pressure.

### 3.3 Analysis of factors associated with the development of hypertension grade ≥3

Table 3 shows the results of both univariate and multivariate analyses of potential predictors of the development of hypertension grade ≥3 during lenvatinib treatment. In the univariate analysis, patients with high‐normal baseline blood pressure were at a greater risk of developing hypertension grade ≥3 than those with optimal baseline blood pressure (odds ratio [OR] = 6.32 [1.61–24.74], *p* = 0.008). In the multivariable analysis, patients classified as having normal or high‐normal baseline blood pressure were at a higher risk of developing hypertension grade ≥3 than those with optimal baseline blood pressure (OR = 5.07 [1.09–23.54], *p* = 0.038 and OR = 7.48 [1.67–33.51], *p* = 0.008, respectively).

**TABLE 3:** Logistic regression analysis of factors associated with the incidence of hypertension grade ≥3.

Variables	Univariate		Multivariate	
	OR (95% CI)	*p* value	OR (95% CI)	*p* value
Sex (female)	2.54 (0.84–7.61)	0.096	3.35 (0.93–11.99)	0.063
Age (≥65 years)	0.24 (0.05–1.19)	0.081	0.22 (0.04–1.22)	0.084
Obesity (BMI ≥25 kg/m^2^)	1.64 (0.46–5.83)	0.445		
eGFR <60 mL/min/1.73 m^2^	1.64 (0.46–5.83)	0.445		
Diabetes	0.68 (0.19–2.38)	0.548		
Baseline blood pressure group				
Optimal (SBP <120 and DBP <80 mmHg)	Reference		Reference	
Normal (SBP 120–129 and/or DBP 80–84 mmHg)	3.57 (0.87–14.64)	0.077	5.07 (1.09–23.54)	0.038
High‐normal (SBP 130–139 and/or DBP 85–89 mmHg)	6.32 (1.61–24.74)	0.008	7.48 (1.67–33.51)	0.008

Abbreviations: BMI, body mass index; CI, confidence interval; DBP, diastolic blood pressure; eGFR, estimated glomerular filtration rate; OR, odds ratio; SBP, systolic blood pressure.

**FIGURE 3:**
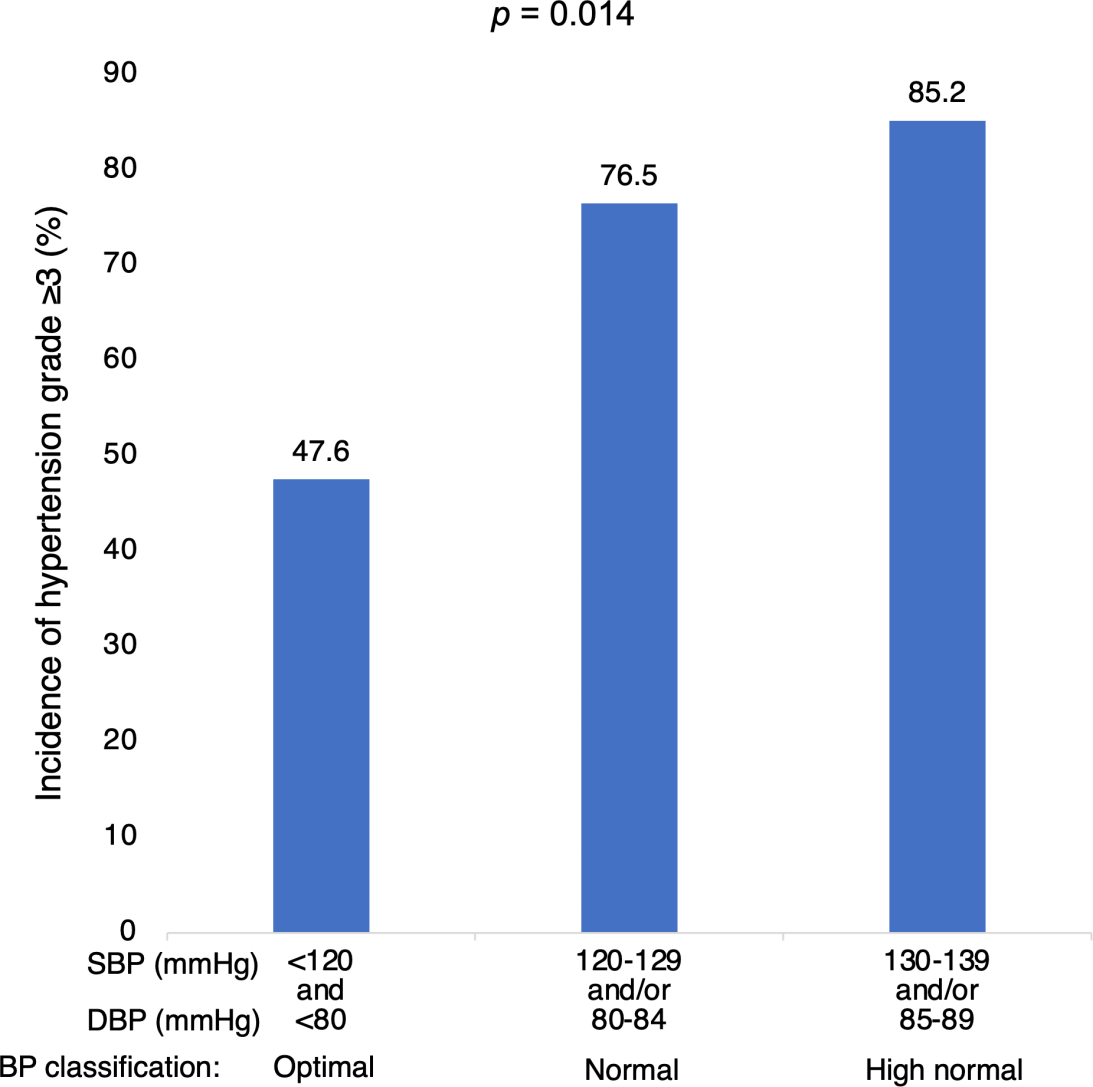
Incidence of hypertension grade ≥3 according to baseline blood pressure classification. *p* = 0.014 by a chi‐squared test. DBP, diastolic blood pressure; SBP, systolic blood pressure.

Figure 3 shows the incidence of hypertension Grade ≥3 based on the baseline blood pressure. There was a significant difference in the incidence of hypertension Grade ≥3 among the three groups (*p* = 0.014). Even patients with normal baseline blood pressure had a 28.9% increase in the incidence of hypertension Grade ≥3 when compared to those with optimal baseline blood pressure. Moreover, in the patients with high‐normal baseline blood pressure, there was a 37.6% increase in the incidence of hypertension Grade ≥3 compared with those with optimal baseline blood pressure.

### 3.4 Antihypertensive therapy

**FIGURE 4:**
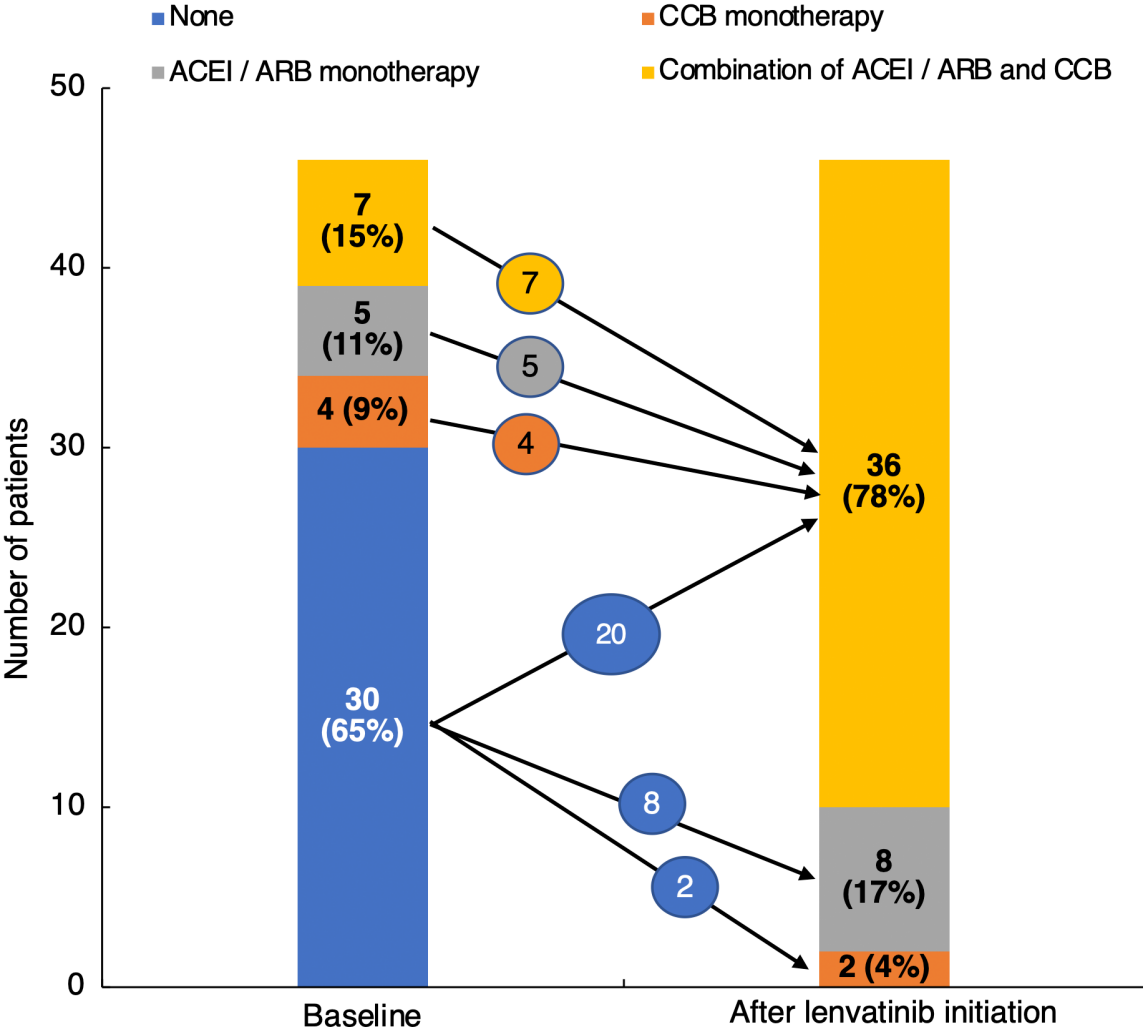
Changes in antihypertensive treatment. ACEI/ARB, angiotensin‐converting enzyme inhibitor/angiotensin II receptor blocker; CCB, calcium channel blocker.

As shown in Figure 2 and Table 2, blood pressure values were controlled equivalently after 1 and 2 weeks of lenvatinib initiation. Among the 65 patients, 46 (71%) required the initiation or intensification of antihypertensive treatment (37 patients [80%] in the HTN group and 9 patients [47%] in the non‐HTN group). Thirty‐six of 46 (78%) patients required a combination of angiotensin‐converting enzyme inhibitor (ACEI)/angiotensin II receptor blocker (ARB) and calcium channel blocker (CCB) to control blood pressure (Figure 4), while 10 patients (22%) were treated with ACEI/ARB or CCB monotherapy. The median (range) time of initiation of antihypertensive intervention was 3 (1–11) days after starting lenvatinib.

## 4 DISCUSSION

In this study, we investigated the actual blood pressure changes with lenvatinib and risk factors for the development of hypertension grade ≥3. The results showed that both SBP and DBP increased on Day 1 after lenvatinib initiation, with a median time to onset of hypertension grade ≥3 of 2 days. In addition, normal blood pressure and high‐normal blood pressure at baseline were found to be significantly associated with an increased risk for the development of hypertension grade ≥3. A previous study showed that hypertension occurred early in the course of lenvatinib treatment, and 47% of the patients treated with lenvatinib experienced their first occurrence of hypertension during cycle 1 of treatment.^16^ Moreover, in Japanese patients, increased blood pressure was observed in the early phase of lenvatinib treatment. If hypertension grade ≥3 develops, treatment should be interrupted and resumed with a reduced dose of lenvatinib after improvement. Therefore, it is important to control the early increase of blood pressure that occurs during lenvatinib induction in order to avoid reducing the dose intensity.

In the present study, lenvatinib caused hypertension grade ≥3 in 71% of patients, similar to the incidence in the Japanese population in the SELECT study.^10^ The mechanism of lenvatinib‐induced hypertension was the inhibition of VEGF in vascular endothelial cells, especially VEGF‐2, which is also the most important mediator of tumor angiogenesis.^19^, ^20^, ^21^ Moreover, compared to vandetanib and sorafenib, which are used for thyroid cancer, lenvatinib has a lower half maximal inhibitory concentration for VEGF‐2 and was reported to have a higher inhibitory activity against VEGF‐2. Therefore, lenvatinib is believed to cause hypertension more frequently than vandetanib and sorafenib.^22^

One case report described the treatment of a patient with thymic carcinoma using the same dose of lenvatinib as for thyroid cancer; the patient developed hypertension grade ≥3 the day after initiating lenvatinib and had difficulty managing his blood pressure.^23^ Similar to this case report, both hypertensive and non‐hypertensive patients in this study had a rapid increase in blood pressure on the day after treatment compared to the blood pressure before lenvatinib administration, with SBP and DBP increasing by approximately 20 and 13 mmHg, respectively. In addition, with regards to the ΔSBP and ΔDBP trends, the maximum value during the treatment period was observed 3 days after the start of treatment in the HTN group. Interestingly, in a recent cohort study evaluating a series of patients treated with doses ranging from 4 to 24 mg/day, the incidence and grade of hypertension were positively correlated with the lenvatinib dosage.^24^ Similarly, higher plasma concentrations of lenvatinib have been reported to be associated with a higher incidence of severe hypertension.^25^ Thus, blood pressure should be carefully monitored during lenvatinib administration because of the highest dose of 24 mg/day for thyroid cancer.

The 2018 ESC/ESH guidelines recommend treatment with antihypertensive drugs when SBP ≥140 mmHg and/or DBP ≥90 mmHg.^17^ According to this criterion, patients with high‐normal blood pressure are usually not eligible for antihypertensive treatment. However, in the present study, 76% of patients with normal blood pressure and 85% of patients with high‐normal blood pressure developed hypertension grade ≥3. In addition, the incidence of grade ≥3 hypertension was 28% and 37% higher in normal blood pressure and high‐normal blood pressure patients, respectively, than in patients with optimal blood pressure. In both HTN and non‐HTN groups, both SBP and DBP increased from baseline to Day 1 after the start of lenvatinib (Figure 2B), but ΔSBP and ΔDBP did not differ significantly between the two groups. This suggests that the incidence of hypertension grade ≥3 increases with baseline blood pressure. Moreover, risk factors for elevated blood pressure have been reported to include high blood pressure, chronic kidney disease, advanced age, obesity, and diabetes mellitus.^26^, ^27^ In this study, the results of the multivariate analysis revealed that normal and high‐normal blood pressure were significantly associated with the development of hypertension grade ≥3. These results suggest that it is important to control baseline blood pressure to SBP <120 and DBP <80 mmHg before initiating lenvatinib, and this may prevent treatment interruption due to hypertension.

In this study, no patient required lenvatinib treatment interruption due to hypertension during the study period. In patients who received antihypertensive treatment intervention, the median time to the initiation of antihypertensive intervention was 3 days after lenvatinib initiation. Thus, it is possible that early antihypertensive intervention prevented blood pressure elevation during the subsequent lenvatinib treatment period during the study period, leading to no requirements for dose reductions or interruptions due to hypertension. Currently, there is no clear evidence to support the use of a combination of ACEI/ARB and CCB as a method of blood pressure control during the use of antitumor drugs with VEGF inhibitory action. However, it has been reported that a combination of antihypertensive drugs with different mechanisms of action provides better antihypertensive effects than increasing the dose of one antihypertensive agent to control blood pressure.^16^ In the present study, 78% of patients who received antihypertensive intervention after starting lenvatinib received a combination of ACEI/ARB and CCB, and no patient had to discontinue lenvatinib due to poorly controlled hypertension. In a recent retrospective cohort study of 29 patients treated with lenvatinib, 95% of patients who were treated with antihypertensive drugs received two or more antihypertensive agents in combination to control blood pressure.^24^ Therefore, after initiation of lenvatinib, early treatment with antihypertensive drugs, including combination drugs, may be useful for good blood pressure control.

It is important to continue treatment for thyroid cancer without decreasing the dose of therapy as much as possible due to the limited treatment options available. A previous report showed that lenvatinib‐induced hypertension was significantly correlated with improved clinical outcomes in patients.^16^ In addition, it has also been reported that a shorter duration of interruption of treatment with lenvatinib improves survival, and higher doses of treatment result in a reduction in tumor size.^12^, ^28^ Therefore, in order to avoid dose reduction or interruption of lenvatinib, it is important to control blood pressure in patients with baseline hypertension by administering antihypertensive treatment before starting lenvatinib, and, in patients with elevated blood pressure after lenvatinib administration, by administering antihypertensive treatment as soon as possible. Active blood pressure control is important not only in the initial treatment with high doses but also in subsequent outpatient treatment, as patients on reduced dosages of lenvatinib of 20 mg/day or 14 mg/day also tended to require interruption or reduced doses of lenvatinib because of hypertension.

Two important limitations of this study must be considered. First, the timing of blood pressure measurements differed because of the retrospective nature of this study, which could have affected blood pressure trends. Second, the timing of antihypertensive treatment initiation and dosage were at the discretion of the physician, and judgments based on the physician's experience and knowledge may have affected the antihypertensive effect. However, as per the results of this study, it is during the early period of treatment with high doses that hypertension is difficult to manage. Furthermore, early initiation of antihypertensive treatment for hypertension with lenvatinib allowed blood pressure management without treatment interruption, which is a significant result of this study.

In conclusion, blood pressure increased from Day 1 after starting lenvatinib, and higher baseline blood pressure was a significant risk factor for developing hypertension grade ≥3. Early initiation of antihypertensive therapy, including combination drugs, may allow for better control of blood pressure, thus preventing treatment discontinuation and maintaining the therapeutic intensity of lenvatinib.

## AUTHOR CONTRIBUTIONS

**Yuma Shibutani:** Conceptualization (equal); data curation (lead); formal analysis (lead); investigation (lead); visualization (lead); writing – original draft (lead). **Kazuko Tajiri:** Conceptualization (equal); formal analysis (supporting); supervision (lead); writing – original draft (supporting); writing – review and editing (lead). **Shinya Suzuki:** Conceptualization (supporting); writing – review and editing (supporting). **Tomohiro Enokida:** Conceptualization (supporting); writing – review and editing (supporting). **Atsunobu Sagara:** Conceptualization (supporting); writing – review and editing (supporting). **Susumu Okano:** Writing – review and editing (supporting). **Takao Fujisawa:** Writing – review and editing (supporting). **Fumiaki Sato:** Writing – review and editing (supporting). **Tetsuro Yumoto:** Writing – review and editing (supporting). **Motohiko Sano:** Writing – review and editing (supporting). **Toshikatsu Kawasaki:** Writing – review and editing (supporting). **Makoto Tahara:** Conceptualization (equal); supervision (equal); writing – review and editing (equal).

## FUNDING INFORMATION

This work was funded by the National Cancer Center Research and Development Fund (2023‐A‐12).

## ETHICS STATEMENT

This study was approved by the review board of the National Cancer Center (research project no. 2022–115).

## PATIENT CONSENT STATEMENT

The requirement of informed consent was waived because this study was conducted according to a retrospective chart review protocol.

## Supporting information


Figure S1:

## Data Availability

The data that support the findings of this study are available from the corresponding author upon reasonable request.
