# Supplementary material for: Association between baseline blood pressure and the incidence of lenvatinib‐induced hypertension in patients with thyroid cancer
Source: Cancer Med. 2023 Oct 30;12(22):20773–82. doi: 10.1002/cam4.6644 (PMC10709743; doi:10.1002/cam4.6644)
Supplement: Supplementary file 1 — Figure S1: [file CAM4-12-20773-s001.docx]

**Supplementary data**

**Supplementary Figure 1:** **Blood pressure trends in patients NOT receiving antihypertensive therapy before lenvatinib treatment**

Abbreviations: BP: blood pressure, HTN: hypertension

**Supplementary Figure 2:** **Blood pressure trends in patients receiving antihypertensive therapy before lenvatinib treatment**

Abbreviations: BP: blood pressure, HTN: hypertension
